# Optimization and assessment of a hybrid geothermal heat pump and wind turbine system in Izmir, Turkey: A 4E analysis approach

**DOI:** 10.1016/j.heliyon.2024.e40975

**Published:** 2024-12-06

**Authors:** Yashar Aryanfar, Jorge Luis García Alcaraz, Julio Blanco Fernandez, Aleksandar G. Georgiev, Ali Keçebaş, Adham E. Ragab, Shabbir Ahmad

**Affiliations:** a Department of Electric Engineering and Computation. Autonomous University of Ciudad Juárez, Av. Del Charro 450 Norte, Col. Partido Romero, Juárez, Chihuahua, Mexico; b Department of Industrial Engineering and Manufacturing, Autonomous University of Ciudad Juárez. Av. Del Charro 450 Norte, Col. Partido Romero, Juárez, Chihuahua, Mexico; c Department of Mechanical Engineering, University of La Rioja, Edificio Departamental, C/San José de Ca-lasanz 31, 26004, Logroño, La Rioja, Spain; d Department of General Engineering, University of Telecommunications and Posts, 1 Akad. Stefan Mladenov Str., 1700, Sofia, Bulgaria; e Department of Energy Systems Engineering, Technology Faculty, Muğla Sıtkı Koçman University, 48000, Menteşe, Muğla, Turkey; f Department of Industrial Engineering, College of Engineering, King Saud University, P.O. Box 800, Riyadh, 11421, Saudi Arabia; g Institute of Geophysics and Geomatics, China University of Geosciences, Wuhan, 430074, China; h Department of Basic Sciences and Humanities, Muhammad Nawaz Sharif University of Engineering and Technology, Multan, 60000, Pakistan

**Keywords:** Hybrid geothermal heat pump, Wind turbine integration, 4E analysis, Energy efficiency, Exergy analysis, Sustainable heating systems

## Abstract

This study explores the optimization and performance of a hybrid energy system combining a geothermal heat pump (GHP) with a wind turbine in Izmir, Turkey. Utilizing a 4E (Energy, Exergy, Economic, and Exergoenvironmental) analysis approach, the system aims to enhance winter heating efficiency. Geothermal heat pumps leverage the Earth's consistent temperatures for heating and cooling, offering a sustainable alternative to traditional energy sources. Key findings include a high coefficient of performance (COP) of 3.916 for the heat pump, a net power output of 118.9 kW, total energy efficiency of 67.93 %, and exergy efficiency of 45.18 %. Economically, the system proves viable with a unit exergy cost of 0.1246 $/kWh, highlighting its cost-effectiveness. The exergoenvironmental analysis demonstrates a factor of 0.8059, indicating a substantial reduction in environmental impact. The integration of geothermal and wind energy demonstrates substantial potential for sustainable energy solutions, crucial for environmental conservation and economic advancement. This comprehensive evaluation highlights the system's operational dynamics, presenting an innovative approach to renewable energy utilization.

## Highlights

• Ground heat pump with wind turbine achieves a COP of 3.916 for efficient heating.
• Energy and exergy efficiencies of the system stand at 67.93 % and 45.18 %.
• Significantly reduces reliance on fossil fuels, aiding environmental conservation.
• Utilizes R134a, optimizing performance for residential heating in Izmir, Turkey.
• Pioneer sustainable energy solutions, enhancing the potential of integrated systems.

## Graphical abstract

**Figure:**
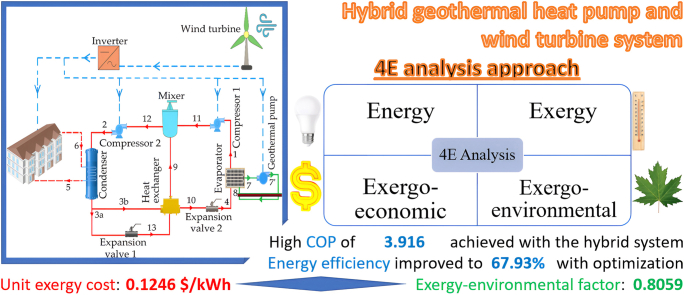

## 1 Introduction

The escalating demand for electrical energy has catalyzed a shift towards renewable sources, broadening the scope of sustainable solutions to encompass heating requirements, historically met through fossil fuel consumption in both industrial and domestic settings [1]. This growing energy demand, coupled with concerns over environmental degradation and the finite nature of fossil fuel resources, presents a significant challenge: the need to develop sustainable, efficient, and low-carbon energy systems. The problem to be solved is how to effectively integrate various renewable energy sources, such as solar, wind, and geothermal, to create systems that are not only environmentally friendly but also economically viable. The pressing need for sustainable and secure power generation systems has intensified due to global energy demand. Renewable resources like solar, wind, and ocean thermal energy are increasingly being explored and utilized to transition from fossil fuels to clean energy sources [2]. Current solutions, such as standalone wind turbines or photovoltaic systems, provide partial answers but often suffer from variability and intermittency issues. Hybrid systems, which combine multiple renewable sources, are emerging as a more robust solution. However, these systems often face challenges related to optimizing performance across different environmental and operational conditions.

Efficient energy mechanisms and greater utilization of renewable resources are crucial for sustainable future energy systems. Recent studies have demonstrated the increasing viability of integrating renewable energy systems, such as wind turbines and photovoltaic panels, to enhance energy production efficiency and reduce dependency on fossil fuels [3]. Notably, Hoseinzadeh et al. [4] have explored the optimization of hybrid energy systems in island environments, showcasing the potential of combining photovoltaic panels and wind turbines to achieve higher energy efficiency. While these hybrid systems show promise, a critical limitation is their dependency on specific geographic and climatic conditions, which can affect their performance and economic viability.

This study seeks to address these limitations by designing and simulating an innovative hybrid system that integrates a Ground Source Heat Pump (GSHP) with a wind turbine. The motivation behind this research is to explore how the combination of geothermal and wind energy can provide a more stable and efficient energy solution for heating and electricity generation, particularly in regions with variable climatic conditions. Central to this transition is the adoption of heat pumps, devices that leverage the thermal gradient between two spaces, akin to the operational dynamics of household refrigerators, to either heat or cool an environment based on seasonal demands. The inherent efficiency of heat pumps, particularly their capacity to repurpose waste heat, marks them as a compelling choice for environmentally conscious and cost-effective climate control [5].

Cooling required for air conditioning is often provided by vapor compression refrigeration cycles, which rely heavily on electrical energy. Recently, low or medium-temperature thermal energy cycles for cooling, such as absorption chillers, have gained attention for their ability to use clean and renewable energy [6]. The convergence of these factors underscores the critical role of heat pumps in the energy paradigm shift, offering a path towards reducing dependency on non-renewable energy sources while enhancing the sustainability and efficiency of heating and cooling systems. The main limitation of current heat pump systems is their dependence on electrical grids, which can be a drawback in regions where grid reliability is an issue. The integration of wind turbines with GSHPs offers a potential solution by providing a local, renewable electricity source that enhances system resilience and reduces grid dependency.

Research into hybrid renewable energy systems has highlighted not only the environmental benefits but also the economic feasibility of such technologies. Hybrid systems that integrate multiple renewable energy sources (solar, wind, ocean thermal energy) offer enhanced efficiency and sustainability, especially in coastal regions. These systems are designed to optimize the use of available renewable resources and improve economic viability through energy management [7,8]. For instance, the integration of wind turbines and GSHPs offers significant cost savings and efficiency improvements, as explored in a techno-economic assessment by Hoseinzadeh and Garcia [9], which underscores the potential for substantial reductions in both energy costs and CO_2_ emissions through optimized hybrid systems.

A GSHP system, recognized for its exceptional efficiency in renewable energy technology, capitalizes on the constant temperature beneath the Earth's surface to facilitate heating and cooling operations within buildings. The exploration of hybrid systems combining geothermal and wind energy sources has become increasingly relevant, particularly in enhancing system resilience and sustainability. Mohammadi et al. [10] examined various configurations of hybrid systems, revealing that the integration of multiple renewable energy sources, including wind turbines and geothermal heat pumps, can significantly optimize energy outputs and system stability. Distinct from other heat pump variants, notably Air Source Heat Pumps (ASHPs), GSHPs exploit the thermal constancy of subterranean environments, which remain largely impervious to atmospheric temperature changes. This characteristic enables GSHPs to efficiently draw heat from the ground during colder periods for heating purposes, and inversely, to dispense excess heat into the ground during warmer seasons, effectuating cooling through a ground heat exchanger network of pipes buried underground. The GSHP's advantage lies in its resistance to external temperature variations, ensuring heightened energy efficiency and steadfast operational performance.

Research into GSHP systems underscores their potential and performance efficacy. Several case studies have validated the practical application of hybrid renewable energy systems in different climatic conditions, demonstrating their adaptability and effectiveness. In a comprehensive study by Hoseinzadeh et al. [11], a hybrid system model incorporating wind turbines and GSHPs was evaluated in an island setting, providing insights into optimizing energy efficiency under varying environmental conditions. Hepbasli [12] detailed a thermodynamic evaluation of a GSHP setup intended for district heating in Turkey, employing a U-bend ground heat exchanger, with findings indicating a heat pump COP of 2.85 and a marginally lower COP for the entire system, yet demonstrating considerable energy conversion and utilization efficiency. Hwang et al. [13] assessed the cooling efficacy of a GSHP in a Korean educational facility, unveiling an average cooling COP of approximately 8.3 and an overall COP of about 5.9 at 65 % partial load, significantly surpassing the ASHP system's efficiency due to a lower condenser temperature in the GSHP system. Lubis et al. [14] embarked on a thermodynamic analysis of a hybrid GSHP system, incorporating a cooling tower for additional heat rejection. Their analysis revealed superior operational performance, with a heating COP of 5.34 and an exergy efficiency of 63.4 %, far exceeding typical air-source systems. Self et al. [15] delved into advanced GSHP configurations with economizers, identifying condenser pressure as a critical factor influencing the system's COP, achieving values up to 6.2, which positions it competitively against alternative heating systems. Pishkariahmadabad et al. [16] pursued an optimization study of a GSHP system with an economizer, exploring the system's dynamics across various working fluids. They pinpointed R134a as the fluid with the highest system COP, emphasizing the importance of fluid choice on the system's energy and exergy efficiency. Zhang [17] tackled the challenges in middle-deep GSHP systems through the development of an all-encompassing unsteady heat transfer model combined with an energy conversion model. This model showcased an optimal circulation rate that significantly augments system performance and highlighted that adjustments in well insulation and thermal conductivity minimally impact the COP. Aryanfar et al. [18] evaluated a GSHP with an economizer, contrasting R134a and R513a as working fluids under varied soil and ambient temperature conditions. Their investigation showed R134a surpassing R513a by approximately 5 % in COP and over 10 % in exergy efficiency, highlighting its superior utility in GSHP applications. Liu et al. [19] provided a comprehensive exergy analysis of GSHPs, emphasizing their efficiency and sustainability when amalgamated with renewable energy sources, illustrating that the exergy efficiency of GSHPs is influenced by factors such as installation depth, system configuration, and heat exchanger characteristics. Further exploration by Zhao et al. [20] into the exergoenvironmental analysis of a GSHP with an intermediate economizer assessed the environmental ramifications of different operational parameters and working fluids. This study revealed that increasing evaporator pressure and superheating levels boosts the system's environmental performance across all examined working fluids. Concurrently, Zhao et al. [21] conducted an experimental investigation on a transcritical CO_2_ GSHP system in Harbin, China, comparing its operational dynamics with conventional heating solutions. The study concluded that the CO_2_ GSHP system drastically reduces emissions and offers the lowest lifecycle cost over a 15-year span compared to traditional boilers. Liu et al. [22] delved into the operational and economic aspects of a district heating scheme integrating deep open-loop GSHP systems and heat pumps, based on practical tests in Hebei, China. The outcomes included pinpointing an optimal mass flowrate ratio for enhanced system efficiency and devising a cost-efficient equipment sizing strategy, primarily influenced by the heat pump's specific cost. Lastly, Ougazzou et al. [23] contrasted GSHP and ASHP systems within Morocco's diverse climatic zones, examining their energy efficiency, economic feasibility, and environmental implications through meticulous simulations. Their findings underscored GSHP systems' superior energy conservation and reduced CO_2_ emissions, presenting them as a viable alternative for sustainable heating and cooling across Morocco. These scholarly contributions profoundly enrich the understanding of GSHP technology, showcasing its environmental benefits, economic potential, and energy efficiency. They collectively advocate for the integration of GSHP systems into sustainable energy solutions, emphasizing the need for careful consideration of operational parameters, system configurations, and working fluid selections for optimal performance enhancements.

The emerging consensus on decarbonization underscores the pivotal role of heat pumps and renewable energy sources as integral components of sustainable energy systems. Strategies for integrating renewable energy technologies are critical to achieving decarbonization goals, especially in remote or isolated regions. Hoseinzadeh and Garcia [9] have highlighted the importance of hybrid systems in island decarbonization efforts, combining photovoltaic and wind energy to reduce grid dependency and enhance local energy autonomy. Notably, heat pumps, functioning on the principle of transferring heat between spaces, emerge as a significant contributor to achieving decarbonization goals, as evidenced by literature [[24], [25], [26]]. However, the reliance of electric heating via heat pumps on renewable sources introduces variability in electricity consumption, influenced by factors such as heat demand, pump efficiency, and external conditions [[27], [28], [29]]. This variability necessitates a nuanced understanding of system dynamics, including the potential for increased peak electricity demands and the consequent need for supplemental dispatchable generation capacity. In addressing these challenges, the integration of renewable energy sources with heat pump systems presents a viable pathway to bolstering system resilience and sustainability. For instance, Ozgener [30] demonstrated the feasibility and thermal efficiency of integrating a geothermal heat pump with a wind turbine for greenhouse heating, highlighting the potential for substantial electricity demand reduction. Similarly, Mehrpooya et al. [31] explored the synergy between geothermal and wind energy in powering an ethylene dichloride cracking unit, achieving notable energy and heat output. This innovation is further exemplified by Aryanfar and Alcaraz [32], who introduced a hybrid system combining a geothermal heat pump with an economizer and wind turbine, revealing significant improvements in system performance through optimal pressure adjustments. The importance of flexible operation and the strategic utilization of thermal storage are emphasized in studies such as Bamisile et al. [33], which showcased a hybrid renewable energy-based multigeneration system, and Li et al. [34], proposing an integrated renewable energy supply system for buildings. Furthermore, Ji et al. [35] investigated wind turbine-driven heat pump systems for residential heating, presenting a compelling case for their adoption in Scotland's push towards net-zero emissions. This body of research collectively illuminates the complex interplay between renewable energy sources, heat pumps, and energy systems, underscoring the need for continued innovation and investigation into optimizing system configurations and enhancing operational flexibility to meet the challenges of decarbonization effectively. These studies contribute to a growing body of evidence supporting the integration of heat pumps with renewable sources as a means to enhance electricity system flexibility and efficiency. Thus, the exploration of heat pump integration with renewable energy sources reveals a promising avenue for advancing decarbonization efforts [36], highlighting both the challenges and innovative solutions that characterize this dynamic field. These studies underscore the potential of renewable-driven heat pump systems in diverse applications, from agricultural to industrial and residential settings, highlighting their role in reducing CO_2_ emissions and improving energy efficiency. In comparison to the previous research by Aryanfar and Alcaraz [32], this study applies a more comprehensive 4E (Energy, Exergy, Economic, and Exergoenvironmental) analysis approach, offering a model that optimizes seasonal heating requirements and improves system performance by accounting for geographical variations, addressing operational and economic enhancements not covered in the prior studies.

Despite the extensive research on GSHP systems, a critical gap remains in the comprehensive evaluation of hybrid systems that integrate GSHPs with other renewable energy sources. Existing studies often focus on specific aspects such as energy efficiency, thermodynamic performance, or environmental impact, but there is a need for a holistic approach that simultaneously addresses multiple performance indicators. This study conducted in Izmir, Turkey, aims to fill this gap by not only embracing the concept of a wind turbine and GSHP hybrid system but also extending the analysis to encompass energy, exergy, economics, and exergoenvironmental impacts (4E). This comprehensive evaluation delineates the system's versatility in addressing heating and electricity needs within a residential context during winter, thus providing a nuanced understanding of its operational dynamics and environmental benefits.

What sets this study apart is its holistic approach to analyzing the hybrid system's performance across four critical dimensions (energy, exergy, economics, and exergoenvironmental impacts) and its focus on the integration of a wind turbine with a GSHP. This combination is relatively unexplored in the existing literature, particularly with such a comprehensive scope of analysis. The study not only corroborates the findings of previous research but also expands the knowledge base by offering novel insights into the optimization and application of hybrid renewable energy systems. The objectives of this study are.

• To design and simulate an innovative hybrid system that incorporates a wind turbine and a GSHP for efficient generation of both heat and electricity.
• To delve into the system's response to changes in key operational parameters like condenser pressure, evaporator pressure, intermediate pressure, ambient temperature, and soil temperature.
• To evaluate the exergy destruction within various components of the system.
• To carry out a comprehensive economic and exergoenvironmental assessment of the proposed hybrid system.

In addition, research questions are.

• How does the integration of a wind turbine with a GSHP impact the overall efficiency and sustainability of the heating and electricity generation system?
• What are the optimal operational parameters for maximizing the efficiency and performance of the hybrid system?
• What are the key factors influencing exergy destruction in the hybrid system, and how can they be mitigated?
• How does the hybrid system perform economically and environmentally compared to conventional systems?

To address these research questions, a detailed methodology was employed. First, a hybrid system model incorporating a wind turbine and a GSHP was designed and simulated using advanced computational tools. The model was subjected to various operational scenarios to understand its performance under different conditions. Main parameters such as condenser pressure, evaporator pressure, intermediate pressure, ambient temperature, and soil temperature were varied systematically to assess their impact on the system's efficiency and performance. Exergy analysis was conducted to evaluate the exergy destruction within different components of the system. Additionally, an economic assessment was performed to determine the cost-effectiveness of the system, and an exergoenvironmental analysis was conducted to evaluate its environmental impacts. This holistic approach ensures a comprehensive understanding of the system's operational dynamics and provides insights into optimizing the performance of hybrid renewable energy systems.

By addressing these questions and employing a robust methodology, this research underscores the innovative potential of hybrid systems in contributing to sustainable energy solutions, marking a significant advancement in the field of renewable energy technology. This study aims to provide a comprehensive framework for the development and implementation of hybrid GSHP systems, emphasizing their role in enhancing energy efficiency, reducing environmental impact, and supporting the transition to sustainable energy systems.

The paper is structured as follows: Section 2 discusses the materials and methods used in the study, including system modeling and simulation setup. Section 3 presents the results of the energy, exergy, economic, and exergoenvironmental analyses. Section 4 provides a discussion of the findings, comparing them with existing literature and highlighting the potential for future research. Finally, Section 5 concludes the paper by summarizing the key outcomes and implications for sustainable energy systems.

## 2 Materials and methods

Today, in addition to the analysis of cycle efficiency, environmental effects and their role in driving up the cost of cycles have also been investigated because of the widespread concern about the increase in Earth's temperature and the significance of the environment under the influence of pollution from fossil fuels. Therefore, the simultaneous study of energy, exergy, thermoeconomics, and exergoenvironmental, or 4E analysis, is required for the design and optimization of thermodynamic cycles. The effectiveness of the first and second laws of thermodynamics and the plan's effects on the economy and environment were examined in the 4E study. The comprehensive optimization of diverse thermodynamic cycles, frequently employed in current research, is one of the key uses of such studies.

### 2.1 System modeling and operational overview

**Fig. 1:**
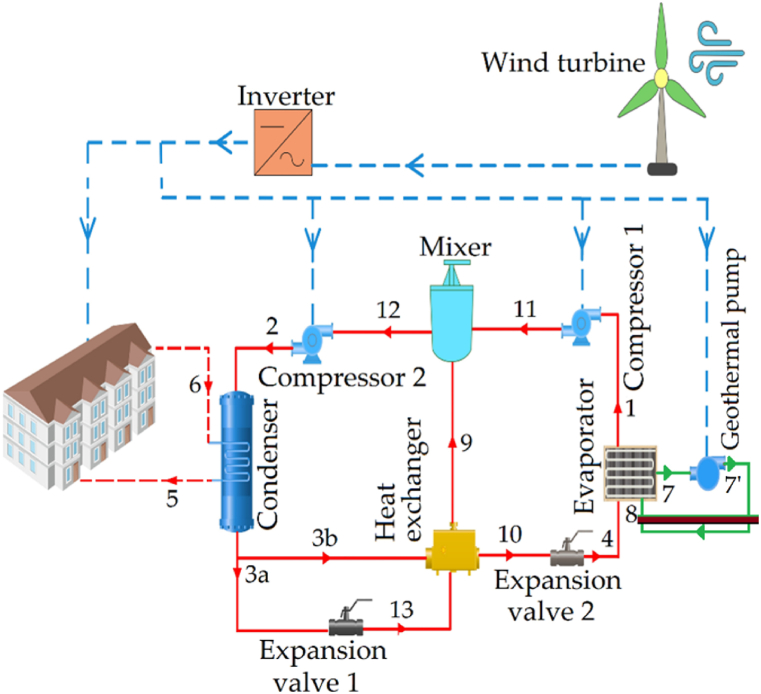
Schematic diagram of the system.

Fig. 1 depicts a GHP system with an intermediate economizer and wind turbine as an integral part of the system. The wind turbine not only supplied energy to the GHP but also fulfilled the household consumption requirements. Any excess electricity generated was fed into the grid. The system initiates with the geothermal evaporator at point 1, where the refrigerant (R134a) is pre-heated before it arrives at the evaporator at point 4. Here, it transitions into high-temperature steam through vaporization. This steam then moves into the compressor, where it undergoes pressure enhancement and superheating at a medium pressure. The streams exiting from Compressor 1 and the heat exchanger merge at point 11 in the mixer, thus completing the circuit. As highlighted in Ref. [37], synchronizing the thermodynamic conditions at points 9 and 11 is crucial for ensuring the proper alignment of the mixer's output with the input of Compressor 2 (point 12), whether the state is saturated or superheated. Compressor 2 processes the mixed current, elevating its pressure to reach point 2, known as the cycle's top condenser. Here, heat discharge occurs to warm the ventilated area (points 5 and 6) at 35 and 30 °C. The output from the condenser is divided into two parts at point 3, where the refrigerant is split. One portion of the fluid (point 3a) flows to Pressure Expansion Valve 1, where the pressure is reduced through an isenthalpic process until it reaches the cycle's intermediate pressure (point 13). Another portion (point 3b) continues through the heat exchanger (main stream), heating the intake to the mixer and the outlet of Pressure Expansion Valve 1 by increasing the temperature below the refrigerant at the exit (point 10). The fluid from the heat exchanger at point 10 then passes through Pressure Expansion Valve 2 and enters the evaporator, where it reaches the cycle's lower pressure. Geothermal fluid at point 7, with a temperature of 11 °C and a flow rate of 4.432 kg/s, is used to supply heat to the heat pump evaporator.

The thermodynamic evaluation of a ground source heat pump system, with a focus on performance coefficients and exergy efficiency, was conducted using the principles of the first and second laws of thermodynamics. This assessment encompasses various components within the cycle and utilizes the commonly used organic fluid R134a in the context of steam compression cycles. The investigation employs the principles of the first and second laws of thermodynamics. The first law focuses on quantifying the energy exchanges occurring within the system, while the second law provides an assessment of the energy exchange quality. System performance degradation is often a consequence of irreversibilities present in various segments of the thermodynamic cycle. In practical scenarios, entropy generation is a result of both external and internal influences. Within the cycle of the heat pump, internal entropy generation is primarily due to friction-induced pressure losses in the piping and heat transfers arising from temperature differentials in the system's components. External entropy generation, on the other hand, is linked to heat transfer processes that extend beyond the system's control volume boundaries. When evaluating the wind turbine's efficiency, the Rankine-Froude Actuator Disc theory [[38], [39], [40]] is utilized to characterize the wind profile through the turbine's rotor-swept area. This analysis also incorporates a set of foundational assumptions for accurate system modeling.

• The subsurface soil was assumed to exhibit uniform characteristics, and the thermal properties of all materials were considered constant over the investigated temperature range.
• The ground temperature is kept constant above and below the boreholes, while the contact resistance between the earth and holes is ignored.
• The system was assumed to function under steady conditions, and the vertical heat conductivity of the soil was not considered.
• The compressors and pumps were treated as adiabatic, and the pressure drops in the heat exchangers and connecting pipes were considered insignificant.
• An ethanol mass proportion of 30 % in water was used to simulate geothermal fluid as a mixture of water and ethanol.
• The wind flow is considered to have a uniform flow velocity inside the disk and is homogenous and incompressible.
• The wind flow passing through the disk is believed to be non-rotating because there are no obstacles in the way the wind flows either upstream or down-stream of the rotor.

**Fig. 2:**
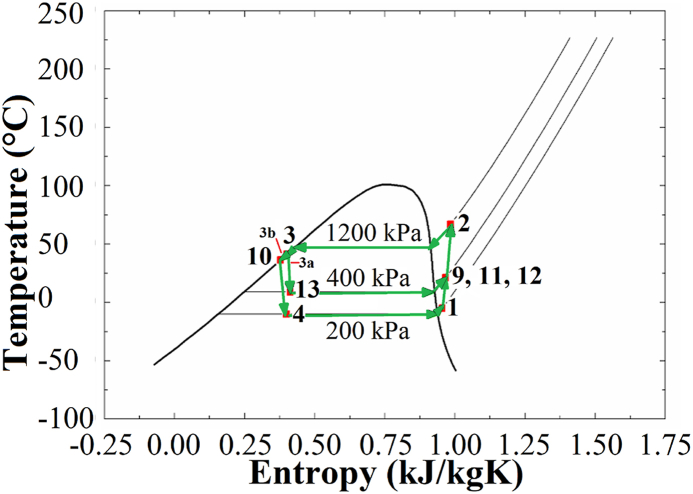
Temperature-entropy diagram of heat pump.

Fig. 2 provides a visualization of the temperature-entropy diagram corresponding to the heat pump section. The operational foundation of heat pumps relies on a core physical attribute: the modulation of the boiling point of a fluid in response to varying pressure levels. By adjusting the pressure, the refrigerant can undergo vaporization at lower temperatures or, conversely, exhibit a higher boiling point. A heat pump or heat pump system constitutes a refrigeration cycle that is engineered to facilitate controlled heat transfer in a specific direction. Consequently, the roles of the evaporator and condenser within this refrigeration cycle interchange dynamically, contingent upon the prevailing seasonal conditions. This versatility renders the system particularly cost-effective in regions where electricity costs are more favorable than those of fossil fuels, making it an appealing choice. Notably, conventional air-conditioning systems can be adapted to function as heat pumps or heat pump systems. Leveraging the heat-pump mechanism allows for the generation of hot water, cold water, or hot and cold air, as dictated by seasonal requirements, without necessitating a separate boiler. This dual functionality not only reduces the initial installation expenses but also optimizes space utilization within the facility's machinery room. Furthermore, this system contributes to a decrease in fossil fuel consumption, thereby mitigating air pollution. Additionally, it enhances building safety, particularly in scenarios involving potential hazards such as fires.

### 2.2 Energy and exergy analyses

The energy balance and irreversibility connection inside each cycle component can be stated as follows (Equation (1) and (2)), according to Refs. [12,41], given the presumption of constant flow circumstances and the assumption that each cycle component serves as the control volume:

(1) $$\dot{Q}-\dot{W}+\sum {\dot{m}}_{in}h_{in}+\sum {\dot{m}}_{out}h_{out}=0$$

(2) $$\dot{I}=\sum \left(1-\frac{T_{0}}{T_{k}}\right){\dot{Q}}_{k}-\dot{W}+\sum {\dot{m}}_{in}\psi_{in}-\sum {\dot{m}}_{out}\psi_{out}$$

and the specific exergy (ψ) and exergy rate are given as

(3) $$\psi =\left(h-h_{0}\right)-T_{0}\left(s-s_{0}\right)$$

The variables $\dot{Q}$, $\dot{W}$, $\dot{m}$, and h stand for the rates of heat exchange, work exchange, mass flow rate, and specific enthalpy, respectively, in the context at hand. The subscripts ‘in’ and ‘out’ refer, respectively, to the input flows into and output flows from the control volume. Irreversibility, ambient temperature, specific entropy, and time are each represented by the variables $\dot{I}$, $T_{0}$, s, and t. In Equation (3), $T_{k}$ stands for the heat source's temperature, and ${\dot{Q}}_{k}$ stands for the heat transfer rate between the heat source and the working fluid per unit mass.

To further calculate the exergy efficiency ($\eta_{ex}$) and the exergy destruction fraction ($y_{ex}$) in different components of the cycle, they are denoted as (Equation (4) and (5)) [12,20], respectively:

(4) $$\eta_{ex,i}=\frac{{\dot{Ex}}_{desired,out}}{{\dot{Ex}}_{used}}$$

(5) $$y_{ex,i}=\frac{{\dot{I}}_{i}}{{\dot{I}}_{tot}}$$

where, $\eta_{ex}$ represents the exergy efficiency, ${\dot{Ex}}_{desired,out}$ corresponds to the exergy related to the desired output currents, and ${\dot{Ex}}_{used}$ is the exergy associated with the input supply currents. Utilizing the balance equations mentioned, the energy, and exergy balance equations, along with the exergy efficiencies for the components depicted in the system flow diagram of Fig. 1, have been enumerated in Table 1.

**Table 1:** Energy and exergy balance equations for the considered system and its components.

Components, i	Energy balance equations	Exergy balance equations	Exergy efficiency
Ground source (GS)	${\dot{m}}_{7^{\prime }}h_{7^{\prime }}+{\dot{Q}}_{GS}={\dot{m}}_{8}h_{8}$	${\dot{m}}_{7^{\prime }}\psi_{7^{\prime }}+\left(1-\frac{T_{0}}{T_{soil}}\right){\dot{Q}}_{GS}={\dot{m}}_{8}\psi_{8}+{\dot{I}}_{GS}$	$\eta_{ex,GS}=\frac{{\dot{m}}_{8}\psi_{8}-{\dot{m}}_{7^{\prime }}\psi_{7^{\prime }}}{\left(1-\frac{T_{0}}{T_{soil}}\right){\dot{Q}}_{GS}}$
Geothermal pump (gl-pump)	${\dot{m}}_{7}h_{7}+{\dot{W}}_{gl-pump}={\dot{m}}_{7^{\prime }}h_{7^{\prime }}$	${\dot{m}}_{7}\psi_{7}+{\dot{W}}_{gl-pump}={\dot{m}}_{7^{\prime }}\psi_{7^{\prime }}+{\dot{I}}_{gl-pump}$	$\eta_{ex,gl-pump}=\frac{{\dot{m}}_{7}\psi_{7}-{\dot{m}}_{7^{\prime }}\psi_{7^{\prime }}}{{\dot{W}}_{gl-pump}}$
Evaporator (eva)	${\dot{m}}_{4}h_{4}+{\dot{Q}}_{eva}={\dot{m}}_{1}h_{1}$	${\dot{m}}_{4}\psi_{4}+{\dot{m}}_{8}\psi_{8}={\dot{m}}_{1}\psi_{1}+{\dot{m}}_{7}\psi_{7}+{\dot{I}}_{eva}$	$\eta_{ex,eva}=\frac{{\dot{m}}_{1}\psi_{1}-{\dot{m}}_{4}\psi_{4}}{{\dot{m}}_{7}\psi_{7}-{\dot{m}}_{8}\psi_{8}}$
Compressor 1 (comp1)	${\dot{m}}_{1}h_{1}+{\dot{W}}_{comp1}={\dot{m}}_{11}h_{11}$	${\dot{m}}_{1}\psi_{1}+{\dot{W}}_{comp1}={\dot{m}}_{11}\psi_{11}+{\dot{I}}_{comp1}$	$\eta_{ex,comp1}=\frac{{\dot{m}}_{11}\psi_{11}-{\dot{m}}_{1}\psi_{1}}{{\dot{W}}_{comp1}}$
Mixer (mix)	${\dot{m}}_{11}h_{11}+{\dot{m}}_{9}h_{9}={\dot{m}}_{12}h_{12}$	${\dot{m}}_{11}\psi_{11}+{\dot{m}}_{9}\psi_{9}={\dot{m}}_{12}\psi_{12}+{\dot{I}}_{mix}$	$\eta_{ex,mix-}=\frac{{\dot{m}}_{12}\psi_{12}}{{\dot{m}}_{11}\psi_{11}-{\dot{m}}_{9}\psi_{9}}$
Compressor 2 (comp2)	${\dot{m}}_{12}h_{12}+{\dot{W}}_{comp2}={\dot{m}}_{2}h_{2}$	${\dot{m}}_{12}\psi_{12}+{\dot{W}}_{comp2}={\dot{m}}_{2}\psi_{2}+{\dot{I}}_{comp2}$	$\eta_{ex,comp2}=\frac{{\dot{m}}_{2}\psi_{2}-{\dot{m}}_{12}\psi_{12}}{{\dot{W}}_{comp2}}$
Condenser (cond)	${\dot{m}}_{3}h_{3}+{\dot{Q}}_{cond}={\dot{m}}_{2}h_{2}$	${\dot{m}}_{2}\psi_{2}+{\dot{m}}_{5}\psi_{5}={\dot{m}}_{3}\psi_{3}+{\dot{m}}_{6}\psi_{6}+{\dot{I}}_{cond}$	$\eta_{ex,cond}=\frac{{\dot{m}}_{5}\psi_{5}-{\dot{m}}_{6}\psi_{6}}{{\dot{m}}_{2}\psi_{2}-{\dot{m}}_{3}\psi_{3}}$
Expansion valve 1 (ev1)	$h_{3a}=h_{13}$	${\dot{m}}_{3a}\psi_{3a}={\dot{m}}_{13}\psi_{13}+{\dot{I}}_{ev1}$	$\eta_{ex,ev1}=\frac{{\dot{m}}_{13}\psi_{13}}{{\dot{m}}_{3a}\psi_{3a}}$
Expansion valve 2 (ev2)	$h_{10}=h_{4}$	${\dot{m}}_{10}\psi_{10}={\dot{m}}_{4}\psi_{4}+{\dot{I}}_{ev2}$	$\eta_{ex,ev2}=\frac{{\dot{m}}_{4}\psi_{4}}{{\dot{m}}_{10}\psi_{10}}$
Heat exchanger (he)	${\dot{m}}_{3b}h_{3b}+{\dot{m}}_{13}h_{13}+{\dot{Q}}_{he}={\dot{m}}_{10}h_{10}+{\dot{m}}_{9}h_{9}$	${\dot{m}}_{10}\psi_{10}+{\dot{m}}_{9}\psi_{9}={\dot{m}}_{3b}\psi_{3b}+{\dot{m}}_{13}\psi_{13}+{\dot{I}}_{he}$	$\eta_{ex,he}=\frac{{\dot{m}}_{10}\psi_{10}-{\dot{m}}_{3b}\psi_{3b}}{{\dot{m}}_{9}\psi_{9}-{\dot{m}}_{13}\psi_{13}}$

The mass flow rates in the heat pump are as follows (Equation (6), (7), and (8)):

(6) $${\dot{m}}_{\text{ref}}=\frac{{\dot{Q}}_{\text{cond}}}{h_{2}-h_{3}}$$

(7) $${\dot{m}}_{\sup}={\dot{m}}_{ref}\frac{h_{10}-h_{3}}{h_{13}+h_{10}-h_{3}-h_{9}}$$

(8) $${\dot{m}}_{\text{main}}={\dot{m}}_{\text{ref}}\frac{h_{9}-h_{13}}{h_{3}+h_{9}-h_{13}-h_{10}}$$

The variables ${\dot{m}}_{ref}$, ${\dot{m}}_{\sup}$ and ${\dot{m}}_{main}$, respectively, reflect the mass flow rates via the condenser, expansion valve 1, and evaporator in the context at hand [20]. Additionally, the condenser's rate of heat exchange (${\dot{Q}}_{cond}$) (Equation (9)) can be stated as follows:

(9) $${\dot{Q}}_{\text{Cond}}={\dot{m}}_{2}h_{2}-{\dot{m}}_{2}h_{3}$$

In the present investigation, it is postulated that the system capacity is 100 kW (${\dot{Q}}_{cond}$). The operational output of the geothermal pump was determined using Equation (10) [42]:

(10) $${\dot{w}}_{\text{pump},\text{gl}}=\frac{{\dot{V}}_{w,gl}{\Delta P}_{w,gl}}{\eta_{\text{pump}}}$$

The volume flow rate of the fluid in the geothermal ring is represented by ${\dot{V}}_{w,gl}$, while $\eta_{pump}$ denotes the isentropic efficiency of the pump. Additionally, ${\Delta Ρ}_{w,gl}$ is used to denote the pressure drop of the fluid in the geothermal ring. The pressure drop is determined as(Equation (11) and (12)):

(11) $${\Delta P}_{w,gl}=f\left(\frac{l_{gl}\rho_{w,gl}V_{w,gl}}{2d_{i,gl}}\right)$$

(12) $$f={\left(0.79\;\ln\;{Re}_{w,gl}-1.64\right)}^{-2}$$

In the aforementioned equations, $\rho_{w,gl}$ stands for the fluid density, $V_{w,gl}$ for the fluid velocity, and ${Re}_{w,gl}$ for the fluid's Reynolds number inside the geothermal exchanger. The interior diameter and length of the geothermal tube, respectively, are denoted by the variables $d_{i,gl}$ and $l_{gl}$, with the length of the geothermal tube being calculated as(Equation (13)):

(13) $$l_{gl}=\frac{\left(\frac{{\dot{Q}}_{eva}}{N_{b}}\right)}{T_{s}-T_{w,gl}}\left(\frac{1}{\pi d_{i,gl}h_{w,gl}}+\frac{\ln\left(\frac{d_{o,gl}}{d_{i,gl}}\right)}{2k_{gl}}+\frac{F}{U_{s}d_{o,gl}}\right)$$

The following variables are defined in the study at hand: $T_{w,gl}$ is the average fluid temperature inside the geothermal tube, $N_{b}$ and $d_{o,gl}$ are the number and outer diameter of the parallel U-shaped geothermal tubes, $h_{w,gl}$ is the heat transfer related to fluid displacement inside the tube, and $k_{gl}$ is the tube's heat transfer coefficient. The variable F represents the ratio of the maximum load hours to the overall heating hours needed in the ventilated space. Additionally, ${\dot{Q}}_{eva}$ denotes the rate of heat exchange in the evaporator, whereas $T_{s}$ and $U_{s}$ stand for soil temperature and soil heat transfer coefficient, respectively. The coefficient of performance (COP) and energy efficiency for the heat pump and complete system were calculated using Equations (14), (15), (16), (17) [43].

(14) $${COP}_{hp}=\frac{{\dot{Q}}_{cond}}{{\dot{W}}_{comp1}+{\dot{W}}_{comp2}}$$

(15) $${\dot{W}}_{net}=q\left(v\right)-{\dot{W}}_{comp1}-{\dot{W}}_{comp2}-{\dot{W}}_{pump,gl}$$

(16) $$\eta_{\text{en},\text{tot}}=\frac{{\dot{Q}}_{cond}+{\dot{W}}_{net}}{{\dot{Q}}_{geo,in}+{\dot{W}}_{wind}}$$

(17) $$\eta_{ex,tot}=\frac{{\dot{Ex}}_{cond}+{\dot{W}}_{net}}{{\dot{Ex}}_{in,gl}+{\dot{W}}_{wind}}$$

The work rate performed by Compressors 1 and 2 is derived from (Equation (18) and (19)):

(18) $${\dot{W}}_{comp1}={\dot{m}}_{main}\left(h_{11}-h_{1}\right)$$

(19) $${\dot{W}}_{comp2}={\dot{m}}_{ref}\left(h_{2}-h_{12}\right)$$

Technical specifications are typically derived from the power curves provided by the manufacturer, utilizing a fundamental equation that is independent of temperature, pressure, humidity, and air density. These manufacturer-introduced curves often do not align with the actual operating conditions of turbines. As a result, power plants are required to establish a power curve model that accurately reflects real-world conditions to regulate the output of the turbine power. The quadratic polynomial power curve, exponential curve, cubic power curve, and approximation cubic power curve are just a few of the curve types used in the parametric approach to simulate the nonlinear section of the power curve.

The quadratic polynomial power curve approximates power as a function of speed through a second-order polynomial equation, expressed as (Equation (20)) [44]:

(20) $$q\left(v\right)=C_{1}+C_{2}v+C_{3}v^{2}$$

where the following Equations (21), (22), (23) are used to derive $C_{1}$, $C_{2}$ and $C_{3}$ from the data of $v_{ci}$, $v_{r}$ and $P_{r}$:

(21) $$C_{1}=\frac{1}{{\left(v_{ci}-v_{r}\right)}^{2}}\left[v_{ci}\left(v_{ci}+v_{r}\right)-4v_{ci}v_{r}{\left(\frac{v_{ci}+v_{r}}{2v_{r}}\right)}^{3}\right]$$

(22) $$C_{2}=\frac{1}{{\left(v_{ci}-v_{r}\right)}^{2}}\left[4\left(v_{ci}+v_{r}\right){\left(\frac{v_{ci}+v_{r}}{2v_{r}}\right)}^{3}-4v_{ci}v_{r}{\left(\frac{v_{ci}+v_{r}}{2v_{r}}\right)}^{3}-{3v}_{ci}-v_{r}\right]$$

(23) $$C_{3}=\frac{1}{{\left(v_{ci}-v_{r}\right)}^{2}}\left[2-4{\left(\frac{v_{ci}+v_{r}}{2v_{r}}\right)}^{3}\right]$$

When utilizing an exponential power curve to represent the power curve in a variable-speed wind turbine, the nonlinear portion of the power curve can be estimated as (Equation (24)) [45].

(24) $$q\left(v\right)=\frac{1}{2}\rho AK_{p}\left(v^{\beta }-{v_{ci}}^{\beta }\right)$$

where *A* stands for the area that the blades sweep, ρ for the density of the air there, $v_{ci}$ for the cut-in speed, and $K_{p}$ and β are constants. A cubic power curve with set to 3 and assumed to be zero can be produced by simplifying Equation (25). Consequently, the power curve adopts a simpler shape, as demonstrated by Equation (25) [46]:

(25) $$q\left(v\right)=\frac{1}{2}\rho AC_{p,eq}v^{3}$$

In this discussion, $C_{p,eq}$ denotes the constant of the cubic coefficient, commonly referred to as the power factor. The estimated cubic power curve is an approximation of Equation (26), which $C_{p,eq}$ is approximated by its maximum value, denoted as $C_{p,\max}$. Consequently, Eq. (27) [47] represents the nonlinear segment of the power curve.

The cubic coefficient, often known as the power factor, or $C_{p,eq}$, is a constant used in this discussion. Equation (26), where $C_{p,eq}$ is approximated by its maximum value, given as $C_{p,\max}$, yields the estimated cubic power curve. Consequently, the nonlinear portion of the power curve is represented by Equation (27) [47]:

(26) $$q\left(v\right)=\frac{1}{2}\rho AC_{p,\max}v^{3}$$

(27) $$q\left(v\right)=C_{p,\max}{\dot{W}}_{wind}$$

### 2.3 Economic and exergoenvironmental analyses

Equation (28) [48] was utilized for the economic analysis of the system to determine the cost rate per unit of energy production for the entire system:

(28) $${\dot{C}}_{tot}={\dot{C}}_{fuel}+{\sum_{k}\left({\dot{Z}}_{CI}+{\dot{Z}}_{OM}\right)}_{k}$$

In this analysis, the fuel cost rate was considered to be zero because of the utilization of geothermal heat as the heat source, which eliminated the need for fuel. Equations (29), (30) are used to calculate the cumulative costs encompassing the operational, maintenance, and investment costs for each component of the system [48]:

(29) $${\dot{Z}}_{CI,k}+{\dot{Z}}_{OM,k}=\frac{Z_{k}\times \emptyset }{N\times 3600}CRF$$

(30) $${\dot{C}}_{tot}=c_{product}{\dot{W}}_{net}$$

$Z_{k}$ stands for the investment cost of each component, Ø for the maintenance coefficient, N for the number of annual running hours, CRF for the return-on-investment coefficient, and $c_{product}$ for the energy cost per unit. Using reference [32], it is possible to calculate CRF (Equation (31)):

(31) $$CRF=\frac{{i\left(i+1\right)}^{n}}{{\left(i+1\right)}^{n}-1}$$

where i stands for the interest rate and n for the system's useful life. Table 2 lists the investment cost functions of each system component.

**Table 2:** Investment cost functions of different system components [49,50].

Component	Capital cost function
Evaporator and Condenser	$1397\times {\left(A_{Ev\;or\;Cd}\right)}^{0.89}$
Heat Exchanger	$2143\times {\left(A_{H.E.}\right)}^{0.514}$
Pump	$1120\times {\left({\dot{W}}_{pump}\right)}^{0.8}$
Recuperator	$2681\times {\left(A_{Rec}\right)}^{0.59}$
Compressor	$71.1\times \frac{\dot{m}r_{c}}{0.01}\ln\;r_{c}$
Wind turbine	$5000\times {\dot{W}}_{windturbine}$

Exergoenvironmental analysis assesses the system performance from an environmental perspective, with its guiding principles established through evaluations of thermodynamic and ambient conditions. The exergoenvironmental factor is expressed as (Equation (32)) [51]:

(32) $$f_{ei}=\frac{{\dot{Ex}}_{tot.des}}{\sum {\dot{Ex}}_{in}}$$

where, the subscripts in and tot.des represent system input and total exergy destruction, respectively, while ($\dot{Ex}$ denotes the exergy rate. The exergy stability factor is calculated as (Equation (33)) [51]:

(33) $$f_{es}=\frac{{\dot{Ex}}_{tot.des}}{{\dot{Ex}}_{tot.out}+{\dot{Ex}}_{tot.des}+1}$$

where, the subscript tot.out signifies the overall energy destruction within the system. Consequently, the total exergy destruction rate maintains a direct relationship with both the exergoenvironment and the exergy stability factors. The exergoenvironmental effect coefficient is determined by (Equation (34)) [51]:

(34) $$C_{ei}=\frac{1}{\eta_{ex}}$$

where, $\eta_{ex}$ represents the exergy efficiency of the system. Equation (35) illustrates the calculation of the efficacy factor for environmental damage [51].

(35) $$\theta_{ei}=f_{ei}C_{ei}$$

Nevertheless, the influence of the exergoenvironmental improvement can be estimated as (Equation (36)) [51]:

(36) $$\theta_{eii}=\frac{1}{\theta_{ei}}$$

### 2.4 Selection of the working fluid and initial conditions

In this study, the chosen working fluid was R134a. The selection of R134a was based on its favorable thermodynamic characteristics, which make it well-suited for heat pump applications. R134a has a normal boiling point of −26.4 °C and a critical temperature of 101.1 °C, providing effective performance for the system under investigation. Table 3 summarizes the key properties of R134a. R134a was chosen for its thermodynamic efficiency, low global warming potential (GWP), non-flammability, and non-toxicity, aligning with the research's emphasis on environmental sustainability and safety.

**Table 3:** Properties of the refrigerant [52].

Refrigerant	Molecular Formula	Molecular weight (kg/kmol)	Normal Boiling Point (°C)	Critical Temperature (°C)	Critical Pressure (MPa)	Safety class	ODP	GWP
R134a	CH_2_FCF_3_	102	−26.4	101.1	4.059	A1	0	1430

The initial conditions for the simulation, relevant to Izmir in winter, are presented in Table 4. These conditions are crucial for accurately modeling the system's performance. The study leverages the unique climatic conditions of Izmir, Turkey, characterized by a Mediterranean climate with mild winters. The geographical coordinates of Izmir are 38.4192° North latitude and 27.1287° East longitude, positioned on the western coast of Anatolia along the Aegean Sea. The system's simulation and analysis were conducted using the RetScreen program, which allows for a comprehensive evaluation of the system's performance under these specific environmental conditions.

**Table 4:** Initial conditions for Izmir in winter.

Definition	Sign	Amount	Reference
Ambient pressure (kPa)	$P_{0}$	101.2	–
Average ambient temperature in Izmir (winter) (°C)	$T_{0}$	7.9	[53]
Evaporator pressure (kPa)	$P_{1}$	200	[16]
Condenser pressure (kPa)	$P_{2}$	400	[16]
Heat exchanger pressure (kPa)	$P_{9}$	1200	[16]
Soil Temperature (°C)	$T_{\text{soil}}$	15	[54]
Compressor 1 efficiency	$\eta_{\text{comp}1}$	0.8	[16]
Compressor 2 efficiency	$\eta_{\text{comp}2}$	0.8	[16]
Geothermal pump efficiency	$\eta_{\text{geopump}}$	0.9	[16]
Heated water temperature (°C)	$T_{5}$	35	[16]
Entrance water temperature (°C)	$T_{6}$	30	[16]
Geothermal pump entrance temperature (°C)	$T_{7}$	7	[16]
Geothermal pump exit temperature (°C)	$T_{8}$	11	[16]
Condenser capacity (kW)	${\dot{Q}}_{\text{cond}}$	100	[16]
Soil heat transfer coefficient (W/m^2^K)	$U_{s}$	12	[55]
The ratio of the number of hours of maximum load to the total number of hours	F	0.4	[55]
Heat transfer coefficient of the tube (W/mK)	$k_{\text{gl}}$	0.45	[55]
Internal diameter (m)	$d_{i}$	0.0218	[55]
Outer diameter (m)	$d_{o}$	0.0267	[55]
Fluid speed inside the geothermal tube (m/s)	V	1	[55]

The characteristics and specifications of the wind turbine used in the study are listed in Table 5. The combination of the geothermal heat pump and wind turbine is designed to optimize energy efficiency and sustainability, leveraging the local environmental conditions to maximize performance.

**Table 5:** Wind turbine features.

Definition	Sign	Amount	Reference
Turbine diameter (m)	D	34	–
Wind speed in Izmir (m/s)	V	7.9	[56]
Wind turbine efficiency (−)	$\eta_{\text{wt}}$	0.9	[16]
Max efficiency (−)	$C_{\text{pwt}}$	0.59	[16]
Density of air (kg/m^3^)	$\rho_{\text{air}}$	1.23	[16]

## 3 Results and discussion

In this study, the EES software was employed to conduct simulations, encompassing equations related to mass and energy conservation throughout the various stages of the heat pump cycle. Additionally, this study explored the irreversibility relations within the system. To represent the cycle accurately, a subset of fluid parameters was chosen for the simulations, with R134a selected as the working fluid. The system was analyzed at high, medium, and low pressures of 1200, 400, and 200 kPa, respectively, while maintaining a target temperature difference of 5 °C between the condenser and evaporator outputs.

Table 6 provides a thorough summary of the results, including the simulation results and information on the temperature, pressure, and mass flow rate at the various system sites. Table 7 also includes the modeling outcomes for the examined system and provides information on a number of system performance metrics, including the predicted coefficient of performance (COP) of the heat pump and the system's total energy efficiency. The environmental parameters, which are methodically calculated and meticulously documented, are also included in Table 7.

**Table 6:** Properties of the different points of the system.

Point	Fluid	*Temperature (°C)*	*Pressure (bar)*	Mass flow rate (kg/s)
*1*	*R134a*	−5.093	*2*	0.5101
*2*	*R134a*	66.99	*12*	0.5343
*3*	*R134a*	41.29	*12*	0.5343
*4*	*R134a*	−10.09	*20*	0.5101
*5*	*Water*	35	*2.5*	4.782
*6*	*Water*	30	*2.5*	4.782
*7*	*Geo-fluid*	7	*2.5*	4.432
*8*	*Geo-fluid*	11	*2.5*	4.432
*9*	*R134a*	21.1	*4*	0.02421
*10*	*R134a*	36.29	*12*	0.5101
*11*	*R134a*	21.1	*4*	0.5101
*12*	*R134a*	21.1	*4*	0.5343
*13*	*R134a*	8.91	*4*	0.02421

**Table 7:** System output results.

Parameters	Value
${\dot{m}}_{\text{ev}1}$	*0.02421*
${\dot{m}}_{\text{eva}}$	*0.5101*
${\dot{W}}_{\text{comp}1}$	*9.26*
${\dot{W}}_{\text{comp}2}$	*16.28*
${\text{COP}}_{\text{hp}}$	*3.916*
${\dot{W}}_{\text{net}}$	*118.9*
$\eta_{\text{en},\text{total}}$	*67.93*
$\eta_{\text{ex},\text{total}}$	*45.18*
$f_{\text{ei}}$	*0.8059*
$f_{\text{es}}$	*0.4656*
$C_{\text{ei}}$	*2.359*
$\theta_{\text{ei}}$	*1.901*
$\theta_{\text{eii}}$	*0.5261*

**Fig. 3:**
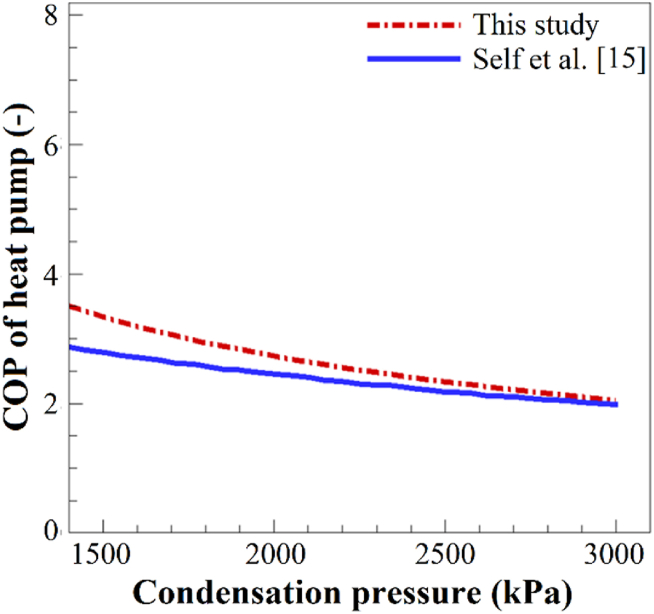
A comparison of the findings of the present study with those of Self et al. [15].

This study compared the impact of high-pressure variations on the coefficient of performance (COP) of the heat pump under identical input conditions with findings reported by Self et al. [15] to validate the present study's results. The validation is depicted in Fig. 3, where an increase in the condensation pressure led to a decrease in the COP. Notably, at lower pressures, such as 1500 kPa, there is a significant deviation in the COP compared to Self et al. [15], but at higher pressures of approximately 3000 kPa, the two sets of results closely align. It's worth highlighting that the standard deviation of the results falls within an acceptable range.

**Fig. 4:**
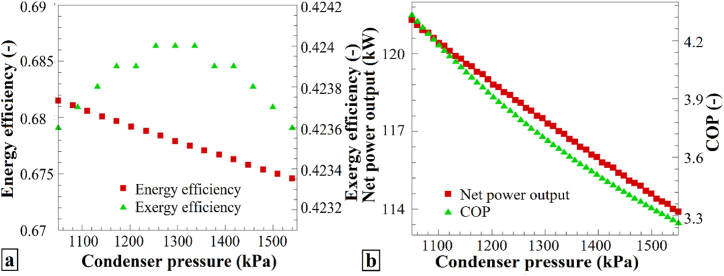
The effects of varying P_2_ on (a) η_en_ and η_ex_, and (b) the Ẇ_net_ and COP of the heat pump system.

The performance parameters for the condenser pressure variations are shown in Fig. 4. Fig. 4a shows the variations in the energy efficiency and exergy efficiency as the condenser pressure ranges from 1050 to 1550 kPa. A higher condenser pressure reduces the energy efficiency owing to the decreased heat transfer efficiency, resulting in less efficient energy utilization, especially at elevated pressures. Conversely, the exergy efficiency initially increases, reaches a peak, and then decreases, indicating an optimal pressure point those balances both the energy and exergy efficiencies. The study identifies the best heat pump system performance occurring at a condenser pressure of approximately 1300 kPa, representing an equilibrium between energy and exergy efficiency.

Fig. 4b complements this analysis by illustrating the changes in the total output work and heat pump performance coefficient in response to condenser pressure adjustments. In both cases, there was a consistent decrease as the condenser pressure varied from 1050 kPa to 1550 kPa. Increasing the condenser pressure decreases both the total output work and the heat pump COP, signifying the system's increased demand for work and reduced energy efficiency at higher pressures. Therefore, an elevated condenser pressure negatively impacts the performance of the heat pump system.

**Fig. 5:**
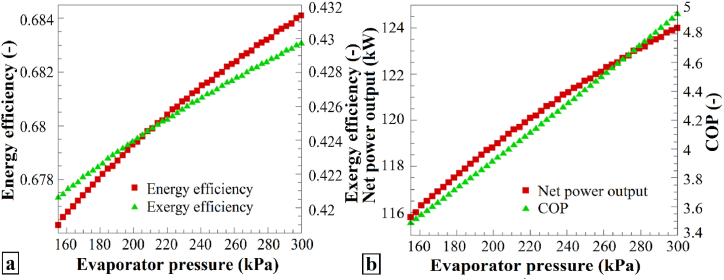
The effects of varying P_1_ on (a) η_en_ and η_ex_, and (b) the Ẇ_net_ and COP of the heat pump system.

The performance parameters for the evaporator pressure variations are shown in Fig. 5. Fig. 5a illustrates the changes in the energy and exergy efficiencies as the evaporator pressure shifts from 155 kPa to 300 kPa. Both the energy and exergy efficiencies improved with increasing evaporator pressure. Consequently, increasing the evaporator pressure positively affects the energy and exergy performance of the system. Fig. 5b further depicts the modifications in the total output work and the heat-pump performance coefficient with changing evaporator pressure. In both cases, an increase was observed when the evaporator pressure increased from 155 to 300 kPa. Thus, increasing the evaporator pressure results in an enhanced total work output and heat-pump performance coefficient.

**Fig. 6:**
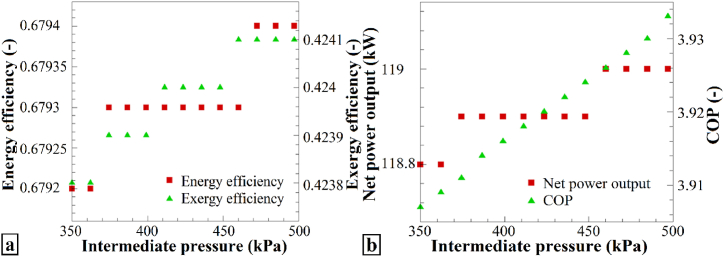
The effects of varying P_9_ on (a) η_en_ and η_ex_, and (b) the Ẇ_net_ and COP of the heat pump system.

Fig. 6 shows the variations in the performance parameters of the intermediate pressure. In Fig. 6a, changes in the energy and exergy efficiencies in response to alterations in the intermediate pressure are displayed. Shifting the condenser pressure from 350 kPa to 500 kPa results in increased energy efficiency and exergy efficiency, indicating that increasing the intermediate pressure has a positive impact on the energy and exergy performance of the system. Fig. 6b presents the modifications in the total output work and the heat-pump performance coefficient with changing intermediate pressure. In both cases, an increase was observed when the intermediate pressure increased from 350 to 500 kPa. Consequently, increasing the intermediate pressure leads to an enhanced total output work and heat-pump performance coefficient.

**Fig. 7:**
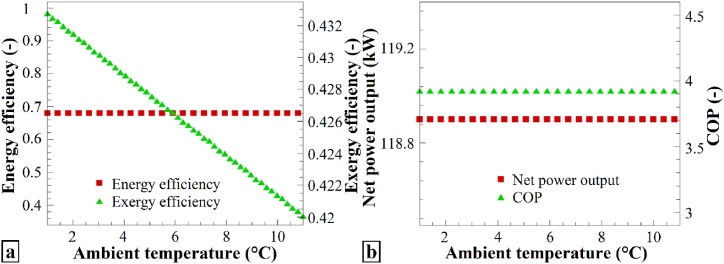
The effects of varying T_0_ on (a) η_en_ and η_ex_, and (b) the Ẇ_net_ and COP of the heat pump system.

Fig. 7 shows the variations in the performance parameters with respect to ambient temperature. Fig. 7a displays the changes in the energy efficiency and exergy efficiency in response to variations in the ambient temperature. As the ambient temperature shifted from 1 °C to 11 °C, the energy efficiency remained unchanged, while the exergy efficiency decreased. Thus, increasing the ambient temperature had no significant impact on the energy performance, and negatively affected the system exergy. Fig. 7b also presents the variations in the total output work and the performance coefficient of the heat pump with changing ambient temperature. Both parameters remained constant when the ambient temperature was changed from 1 °C to 11 °C. Consequently, increasing the ambient temperature did not influence the total work output or the heat pump performance coefficient. Changes in ambient temperature may not have a direct impact on energy efficiency, total output work, or COP, but they can affect other performance parameters, such as exergy efficiency.

**Fig. 8:**
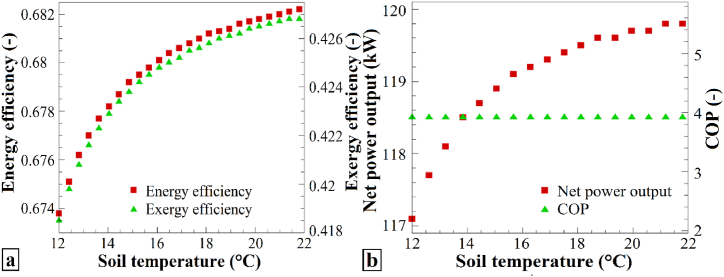
The effects of varying T_soil_ on (a) η_en_ and η_ex_, and (b) the Ẇ_net_ and COP of the heat pump system.

Fig. 8 depicts the variations in the performance parameters in relation to the soil temperature. Fig. 8a illustrates the changes in the energy efficiency and exergy efficiency as the soil temperature changes from 12 °C to 22 °C. Both the energy efficiency and exergy efficiency increase with increasing soil temperature, indicating a positive impact on the energy and exergy performance of the system. Fig. 8b also presents the changes in the total output work and the performance coefficient of the heat pump in response to soil temperature variations. The total work output increased as the soil temperature increased from 12 °C to 22 °C, while the heat pump performance coefficient remained constant. Consequently, an increase in soil temperature has a positive effect on the total output work and does not significantly affect the heat-pump performance coefficient. Soil temperature changes appeared to have a direct influence on energy efficiency, exergy efficiency, and total output work, while the heat pump performance coefficient remained relatively stable.

**Fig. 9:**
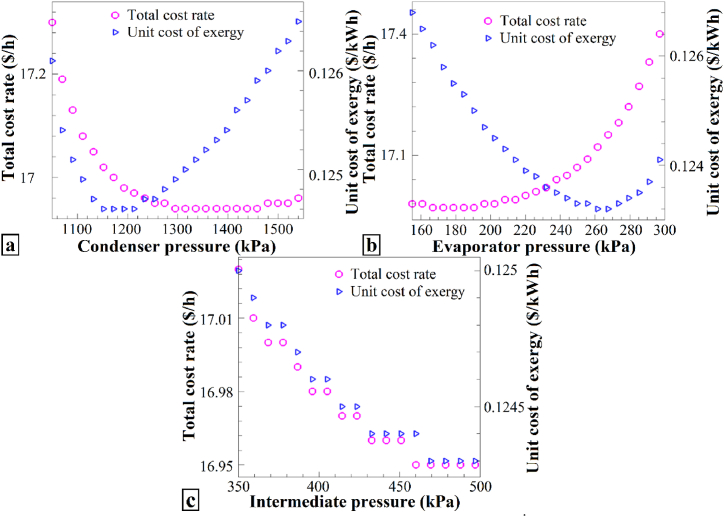
The effects of (a) P_2_, (b) P_1_, and (c) P_9_ on Ċ_tot_ and c_ex_.

Fig. 9 illustrates the impact of changes in the (a) condenser pressure, (b) evaporator pressure, and (c) intermediate pressure on the total cost rate and unit cost of exergy. Across all three scenarios, as the respective pressure values increased (condenser pressure from 1050 kPa to 1550 kPa, evaporator pressure from 155 kPa to 300 kPa, and intermediate pressure from 350 kPa to 500 kPa), both the total cost rate and unit cost of exergy exhibited a consistent pattern: they initially decreased, reached a minimum, and then began to increase. This pattern suggests that the optimal operating point for each parameter corresponds to the minimum point in the graph. Specifically, for the condenser pressure (Fig. 9a), the optimal values are approximately 16.15 $/h for the total cost rate and 0.124 $/kWh for the unit cost of exergy, occurring at approximately 1200 kPa. Similarly, for the evaporator pressure (Fig. 9b), the optimal values are approximately 17.12 $/h for the total cost rate and 0.123 $/kWh for the unit cost of exergy at approximately 180 kPa.

Finally, for intermediate pressure (Fig. 9c), the optimal values are estimated to be approximately 16.95 $/h for the total cost rate and 0.1243 $/kWh for the unit cost of exergy, with a minimum of approximately 470 kPa. The observed patterns in the total cost rate and unit cost of exergy, concerning changes in the condenser pressure, evaporator pressure, and intermediate pressure, consistently demonstrated a behavior where they initially decreased, reached a minimum point, and then increased. This behavior indicates that there exists an optimal operating point for each parameter, represented by the minimum point on the respective graph. Specifically, the optimal condenser pressure appears to be approximately 1200 kPa for the total cost rate and 1400 kPa for the unit cost of exergy. Similarly, the optimal evaporator pressure corresponds to approximately 180 kPa for the total cost rate and 260 kPa for the unit cost of exergy. Finally, the optimal intermediate pressure is estimated to be approximately 470 kPa for the total cost rate and 480 kPa for the unit cost of exergy.

**Fig. 10:**
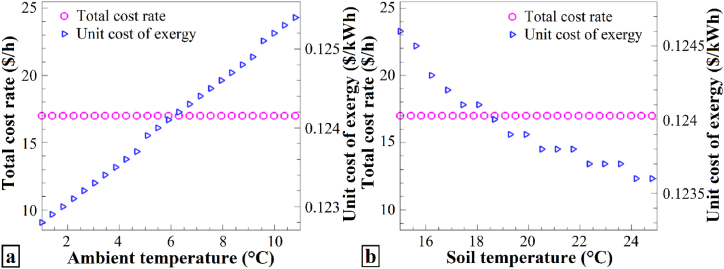
The effects of (a) T_0_, and (b) T_soil_ on Ċ_tot_ and c_ex_.

Fig. 10 shows the impact of (a) ambient temperature and (b) soil temperature changes on the total cost rate and unit cost of exergy. It is evident from Fig. 10 that variations in ambient and soil temperatures did not significantly influence the total cost rate. Specifically, an increase in ambient temperature leads to an increase in income (Fig. 10a), whereas an increase in soil temperature results in a decrease in income (Fig. 10b). In summary, the unit cost of exergy increases with increasing ambient temperature, but decreases with increasing soil temperature. In addition, the unit cost of exergy is influenced by changes in ambient and soil temperatures, with an increase in ambient temperature leading to higher unit exergy costs, whereas an increase in soil temperature results in lower unit exergy costs.

However, these temperature variations do not significantly affect the total cost rate. The unit cost of exergy increases with increasing ambient temperature because higher temperatures lead to higher energy consumption and costs to maintain system performance (Fig. 10a). In contrast, increasing the soil temperature decreases the unit cost of exergy owing to the improved system efficiency, thereby reducing the overall energy consumption and costs (Fig. 10b). Generally, higher ambient temperatures result in higher exergy costs, whereas higher soil temperatures lead to lower exergy costs owing to their respective effects on system performance and efficiency.

**Fig. 11:**
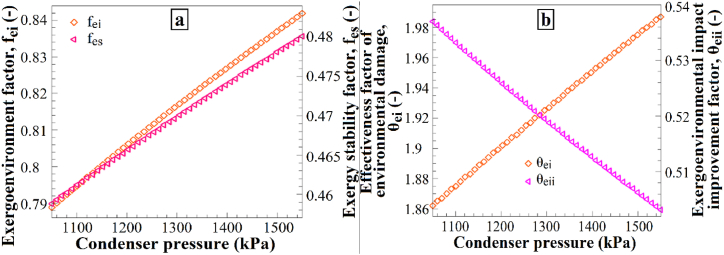
The effects of P_2_ changes on (a) f_ei_ and f_es_, and (b) θ_ei_ and θ_eii_.

Fig. 11 illustrates the impact of condenser pressure variations on (a) the exergoenvironmental factor and exergy stability factor and (b) the effectiveness factor of environmental damage and the exergoenvironmental impact improvement factor. Increasing the condenser pressure from 1050 kPa to 1550 kPa resulted in an increase in the exergoenvironmental factor, exergy stability factor, and effectiveness factor of environmental damage (Fig. 11a), while the exergoenvironmental impact improvement factor decreased (Fig. 11b). This result demonstrates how the change in condenser pressure affects the environmental impacts and exergy stability. The increase in condenser pressure leads to higher values for the exergoenvironment factor, exergy stability factor, and effectiveness factor of environmental damage, possibly because of the higher energy consumption associated with elevated condenser pressure, which contributes more to environmental impacts.

**Fig. 12:**
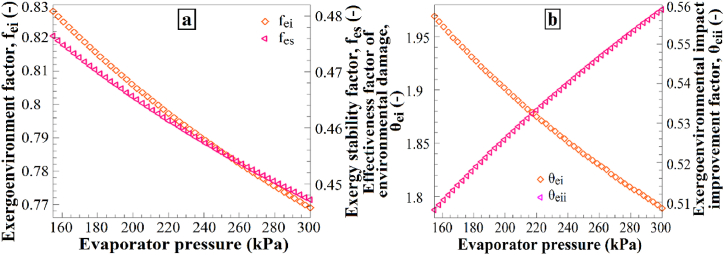
The effects of P_1_ changes on (a) f_ei_ and f_es_, and (b) θ_ei_ and θ_eii_.

Additionally, the decrease in the exergoenvironmental impact improvement factor suggests that a higher condenser pressure may increase the exergy losses within the system, thus amplifying the environmental effects. Therefore, an increase in condenser pressure is expected to influence these factors accordingly. Fig. 12 shows the impact of evaporator pressure variations on (a) the exergoenvironmental factor and exergy stability factor and (b) the effectiveness factor of environmental damage and the exergoenvironmental impact improvement factor. Increasing the evaporator pressure from 155 kPa to 300 kPa resulted in a decrease in the exergoenvironmental factor, exergy stability factor, and effectiveness factor of environmental damage (Fig. 12a), while the exergoenvironmental impact improvement factor increased (Fig. 12b). These results demonstrate how the increase in evaporator pressure affects the environmental effects and exergy stability. The increase in evaporator pressure leads to a decrease in the exergoenvironmental factor, exergy stability factor, and effectiveness factor of environmental damage. This may be attributed to the higher evaporator pressure, which requires less energy consumption in the system, thus contributing less to the environmental effects. Additionally, the increase in the exergoenvironmental impact improvement factor suggests that a higher evaporator pressure can reduce exergy losses in the system, subsequently decreasing the environmental impacts. Therefore, an increase in evaporator pressure is expected to cause changes in these factors.

**Fig. 13:**
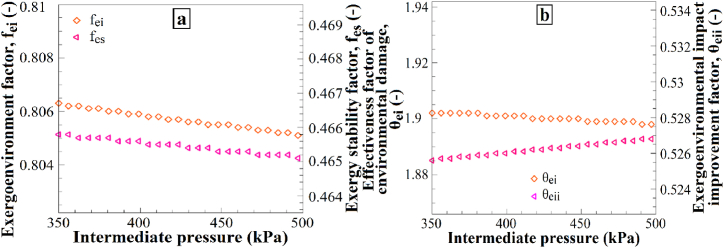
The effects of P_9_ changes on (a) f_ei_ and f_es_, and (b) θ_ei_ and θ_eii_.

Fig. 13 shows the impact of intermediate pressure variations on (a) the exergoenvironmental factor and exergy stability factor and (b) the effectiveness factor of environmental damage and the exergoenvironmental impact improvement factor. Increasing the intermediate pressure from 350 kPa to 500 kPa resulted in a decrease in the exergoenvironmental factor, exergy stability factor, and effectiveness factor of environmental damage (Fig. 13a), while the exergoenvironmental impact improvement factor increased (Fig. 13b). These results demonstrate how an increase in the intermediate pressure affects the environmental impact and exergy stability. The increase in intermediate pressure leads to a reduction in the exergoenvironmental factor, exergy stability factor, and effectiveness factor of environmental damage. This may be attributed to the fact that a higher intermediate pressure results in lower energy consumption in the system, thereby contributing less to the environmental impacts. Additionally, the increase in the exergoenvironmental impact improvement factor suggests that a higher intermediate pressure can reduce exergy losses in the system, consequently mitigating the environmental effects. Therefore, an increase in the intermediate pressure is expected to cause these changes in the mentioned factors.

**Fig. 14:**
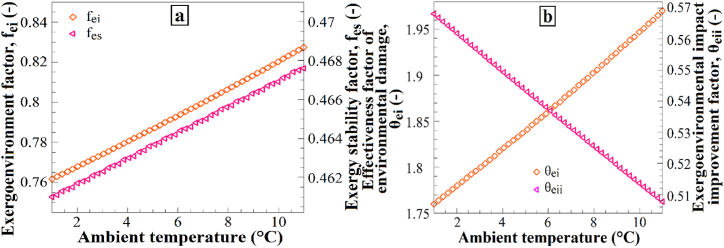
The effects of T_0_ changes on (a) f_ei_ and f_es_, and (b) θ_ei_ and θ_eii_.

Fig. 14 shows the impact of ambient temperature variations on (a) the exergoenvironmental factor and exergy stability factor and (b) the effectiveness factor of environmental damage and the exergoenvironmental impact improvement factor. Increasing the ambient temperature from 1 °C to 11 °C resulted in a decrease in the exergoenvironmental factor, exergy stability factor, and effectiveness factor of environmental damage (Fig. 14a), whereas the exergoenvironmental impact improvement factor increased (Fig. 14b). The observed changes in these factors with varying ambient temperatures reflect the trade-off between the energy consumption and environmental impact. Higher temperatures generally lead to increased energy consumption and, consequently, greater environmental damage, resulting in observed decreases in the exergoenvironmental factor, exergy stability factor, and effectiveness factor of environmental damage. Conversely, lower ambient temperatures reduce the energy consumption and environmental impact, leading to an increase in the exergoenvironmental impact improvement factor.

**Fig. 15:**
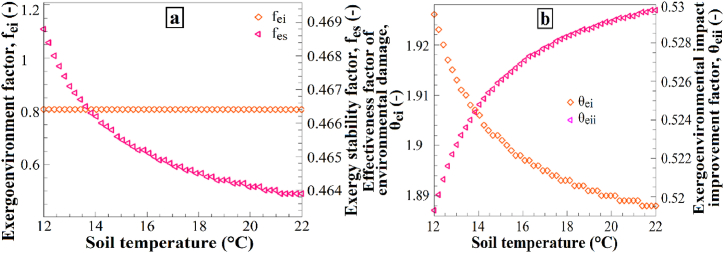
The effects of T_soil_ changes on (a) f_ei_ and f_es_, and (b) θ_ei_ and θ_eii_.

Fig. 15 presents the impact of soil temperature variations on (a) the exergoenvironmental factor and exergy stability factor and (b) the effectiveness factor of environmental damage and the exergoenvironmental impact improvement factor. Increasing the soil temperature from 12 °C to 22 °C resulted in a decrease in the exergoenvironmental factor, exergy stability factor, and effectiveness factor of environmental damage (Fig. 15a), whereas the exergoenvironmental impact improvement factor increased (Fig. 15b). These results demonstrate how an increase in soil temperature affects the environmental impacts and exergy stability. Increasing the soil temperature leads to a reduction in the exergoenvironmental factor, exergy stability factor, and effectiveness factor of environmental damage. This may be attributed to the fact that higher soil temperatures contribute less to environmental impacts owing to lower energy consumption within the system. Additionally, the increase in the exergoenvironmental impact improvement factor suggests that higher soil temperatures can reduce the environmental effects by decreasing exergy losses within the system. Therefore, an increase in soil temperature is expected to bring about these changes in the mentioned factors.

Table 8 provides a comparative overview of various studies that explore the integration of wind and geothermal energy systems, focusing on their efficiency and economic implications. These studies span diverse applications, from hydrogen chloride production and multigenerational systems to renewable energy-supported buildings, demonstrating the versatile potential of combining wind and geothermal sources. The study from Izmir, Turkey, distinctively showcases the high energy and exergy efficiency potential of hybrid heating systems that combine GSHP and wind turbines. With energy and exergy efficiencies reaching 67.93 % and 45.18 % respectively, and a unit exergy cost of 0.1246 $/kWh, this study underscores the technical and economic feasibility of utilizing hybrid renewable energy systems for heating purposes. Compared to the other studies, it presents one of the highest efficiencies, highlighting the effectiveness of GSHP and wind turbine integration in optimizing energy use and reducing environmental impact. This study contributes to understanding the efficiency gains achievable through the synergy of wind and geothermal energy in heating systems, with the developed hybrid system showing a marked improvement in energy efficiency compared to typical systems. Typical systems, such as standalone GSHPs or wind turbines, generally exhibit energy efficiencies ranging between 45 and 55 %, whereas our developed system achieves an energy efficiency of 67.93 %. However, its limitations include a focused scope on heating without broader consideration of other energy needs, such as cooling or power generation for other applications.

**Table 8:** Comparative overview of wind and geothermal combined system studies focusing on efficiency and economics.

System characteristics	City, country	Authors	Performance indicators	Economic evaluation	Main findings
Hydrogen Chloride production via integrated Ethylene Dichloride thermal cracking with the wind turbine, GSHP, and ORC (hydrogen)	Meshkin Shahr, Iran	Mehrpooya et al. [31]	Energetic COP: 1.846; Exergetic COP: 1.148	Cost of the Hydrogen Chloride: 0.0642 US$/kg; Payback period: <6 years	Significant potential for sustainable Hydrogen Chloride production through hybrid system.
Wind-geothermal hybrid multigeneration system (power, hot water, cooling, and hydrogen)	Inner Mongolia, China	Bamisile et al. [33]	Energy Efficiency: 58.6 %; Exergy Efficiency: 39.33 %	Levelized cost of electricity for multigeneration system: 0.0103 $/kWh.	Hybrid system significantly enhances energy efficiency while reducing CO_2_ emissions.
Renewable energy-supported building with wind, photovoltaic/thermal and alkaline fuel cell integration (power, heating, hydrogen and energy storage)	Zhengzhou, China	Li et al. [34]	COP of system: 2.91; COP of HP: 3.82	Life cycle cost of system: $09113.85; Payback period: 8.3 years	Integration of photovoltaic/thermal and alkaline fuel cell shows promising potential for efficient and eco-friendly energy provision in buildings.
Hybrid heating system that combines a GSHP and a wind turbine (power and heating)	Izmir, Turkey	This study	Energy Efficiency: 67.93 %; Exergy Efficiency: 45.18 %	Cost of unit exergy: 0.1246 $/kWh	High energy and exergy efficiency potential of wind turbine integrated GSHP systems.

## 4 Conclusion

This study presents a comprehensive 4E (Energy, Exergy, Economic, and Exergoenvironmental) analysis of a novel hybrid system integrating a Ground Source Heat Pump (GSHP) with a wind turbine for residential heating in Izmir, Turkey. The findings reveal significant advancements in system performance, with the hybrid configuration achieving a high coefficient of performance (COP) of 3.916, an energy efficiency of 67.93 %, and an exergy efficiency of 45.18 %. These results underscore the system's potential for high operational efficiency and environmental sustainability, particularly when compared to conventional systems. For example, typical GSHP systems without wind integration typically achieve a coefficient of COP of around 3.0–3.5, while our hybrid system achieves a COP of 3.916, representing a substantial improvement. This demonstrates the growth in system performance when integrating multiple renewable energy sources. The analysis highlights that optimizing operational parameters, such as condenser and evaporator pressures, is crucial for maximizing efficiency, while stable soil temperatures further enhance performance. Economically, the system proves viable with a unit exergy cost of 0.1246 $/kWh, indicating cost-effectiveness in the long term, defined here as a period spanning 15–20 years. Over this time frame, the investment in the hybrid system is expected to yield significant returns, particularly through reduced energy costs and lower emissions compared to conventional systems. Environmentally, the hybrid system demonstrates reduced exergy losses and lower emissions compared to conventional methods, with potential for further improvement through advanced control strategies and additional renewable integrations. For designers and energy engineers, the study recommends careful optimization of pressure levels, maintaining soil temperature stability, and leveraging local climatic conditions to maximize benefits. The use of R134a as a working fluid is effective, though exploring alternative refrigerants with lower global warming potential is advised. This research underscores the importance of hybrid renewable energy systems in advancing sustainable energy solutions, providing a valuable framework for enhancing energy efficiency, reducing environmental impact, and supporting the transition to greener energy systems.

## CRediT authorship contribution statement

**Yashar Aryanfar:** Writing – review & editing, Writing – original draft, Visualization, Validation, Supervision, Data curation, Conceptualization. **Jorge Luis García Alcaraz:** Writing – review & editing, Visualization, Data curation. **Julio Blanco Fernandez:** Writing – review & editing, Visualization, Software, Methodology, Investigation, Data curation. **Aleksandar G. Georgiev:** Writing – review & editing, Writing – original draft, Validation, Methodology. **Ali Keçebaş:** Writing – review & editing, Writing – original draft, Validation. **Adham E. Ragab:** Writing – review & editing, Software, Investigation, Conceptualization. **Shabbir Ahmad:** Writing – review & editing, Software, Investigation.

## Data availability statement

Data will be made available on request.

## Declaration of competing interest

The authors declare that they have no known competing financial interests or personal relationships that could have appeared to influence the work reported in this paper.
